# Apolipoprotein E polymorphism modulation of asymmetric dimethylarginine in hypertensive patients is determined by renal function

**DOI:** 10.1186/s12944-016-0182-y

**Published:** 2016-01-20

**Authors:** Mauro Sergio Martins Marrocos, Andrei Alkmin Teixeira, Beata Marie Quinto, Silmara de Melo Carmona, Mariana Kuniyoshi, Cassio Jose Rodrigues, Maria Aparecida Dalboni, Silvia Manfredi, Maria Eugênia Canziani, Marcelo Costa Batista

**Affiliations:** Nephrology Division, Universidade Federal de São Paulo, R. Pedro de Toledo, 781 14 .andar, Vila Clementino, São Paulo, CEP 04039-032 Brazil; Nephrology Division-New England Medical Center, Tufts University, Boston, MA USA; Research and Education Institute, Hospital Israelita Albert Einstein, São Paulo, São Paulo Brazil

**Keywords:** Asymmetric Dimethylarginine, Apolipoprotein E, Polymorphism, Atherogenesis, Chronic Kidney Disease

## Abstract

**Background:**

Endothelial dysfunction is considered an early step of atherosclerotic vascular disease. Asymmetric dimethylarginine (ADMA), the main endogenous inhibitor of nitric oxide synthase (NOS), plays a critical role in the process of atherosclerosis in a uremic environment. Increased plasma ADMA not only works as a cardiovascular morbidity biomarker but it is also involved in the genesis of atherosclerosis in renal disease. Considering the relationships of apolipoprotein E(ApoE) polymorphism with LDL cholesterol (LDL-C) levels and coronary risk, it is possible that it brings on susceptibility to endothelial dysfunction and atherogenesis seen on uremia.

**Methods:**

Six hundred twenty patients were stratified according to glomerular filtration rate (GFR) estimated by Chronic Kidney Disease Epidemiology Collaboration (CKDEPI) formula: group I > 60 mL/min, group II ≤ 60 mL/min and > 15 mL/min, and group III ≤ 15 mL/min or in hemodialysis. Polymorphic ApoE analysis was performed by polymerase chain reaction amplification (PCR). Plasma ADMA levels were measured by high performance liquid chromatography (HPLC). Groups were compared on clinical and laboratory characteristics as well as allele and genotype distribution towards.

**Results:**

The ε2 allele of ApoE was present in 62 (10.3 %) patients, ε3 allele in 581 (96.2 %), and ε4 allele in 114 (18.9 %). Their distribution among the 3 groups was uniform. Such uniformity was not observed when we considered endothelial function measured by asymmetric dimethylarginine. In group III, the frequency of ε4 allele was significantly lower in the third tertile compared with the first tertile (14.7 versus 53.3 %, *P* = 0.000; Pearson chi-square). In groups I and II, there was no difference in allele frequency according to ADMA levels. This association remained significant even after confouding factors corrections (OR 0.329, 95 % CI 0.155 - 0.699, *P* = 0.004).

**Conclusions:**

The results of this study shows that the frequency of ε4 allele of ApoE is significantly lower among hypertensive patients on hemodialysis with the highest levels of ADMA. Uremia is capable of determining lower plasma ADMA levels in hypertensive ε4 allele carriers.

## Background

As stated in the 2014 United States Renal Data System Annual Data Report, the burden of cardiovascular disease (CVD) in chronic kidney disease (CKD) is still considerable, where CVD in CKD individuals is 2–3 more frequent when compared with the general population. There is a stepwise decrease in survival related to advanced CKD stage, and mortality after congestive heart failure (CHF) or myocardial infarction (MI) in patients with stages 4–5 CKD is similar to that of dialysis patients [1].

It was first reported in 1992 that nitric oxide (NO) synthesis can be inhibited by ADMA, which is substantially elevated in plasma of patients on hemodialysis. It was suggested that the accumulation of ADMA and concomitant inhibition of NO synthesis may contribute to hypertension, immune dysfunction, and CVD in these patients [2]. ADMA, as the main endogenous inhibitor of NOS, plays a critical role in the process of atherosclerosis in a uremic environment [3]. Endothelial dysfunction resulting from reduced NO activity is considered an early step of atherosclerotic vascular disease [4]. Increased plasma ADMA concentrations not only works as a cardiovascular morbidity biomarker but is causally involved in the genesis of atherosclerosis in renal disease [5–7].

Also related to atherosclerosis, ApoE is a multifunctional protein that plays a major role in the metabolism of cholesterol and triglycerides by binding to receptors in the liver to help mediate the clearance of chylomicrons and very low-density lipoproteins from the bloodstream. ApoE polymorphism is also implicated in the burden of CVD. Boerwinkle et al. reported that up to 17 % of the genetic variability in total plasma cholesterol may be attributable to the ApoE polymorphism [8].

Since ApoE polymorphism is a key player in lipid metabolism, it is possible that it leads to susceptibility to endothelial dysfunction and atherogenesis. Aim of study was to evaluate the interaction between ADMA levels and ApoE polymorphism in patients stratified according 3 different degrees of renal function.

## Results

Demographic and laboratory characteristics of the patients are summarized in Table 1. Eight subjects were excluded because of missing ApoE polymorphism data, leaving 612 subjects for analysis. Mean age of the patients was 60.1 ± 14.1 years, 311 (50.7 %) were male, mean body mass index (BMI) was 28.0 ± 5.3, 386 (63.2 %) were Caucasians, 238 (38.9 %) had Diabetes Mellitus (DM), and 218 (35.6 %) had CVD. Groups I, II and III were composed respectively of 199, 206 and 207 patients. In comparison with group I, groups II and III included more males and Caucasians, while group III had a lower mean age (52.4 ± 14.9 years). Hemodialysis vintage in group III was 37.36 ± 26.84 months. BMI and waist circumference decreased from group I to group III. Prevalence of DM was highest in group II (53.4 %); metabolic syndrome and CVD increased in prevalence from group I to group III. Use of statins was predominate in groups II and III (54.3 % and 53.2 %).

**Table 1:** Demographic and laboratory characteristics of patients stratified by renal function

	Group I (N = 189)	Group II (N = 222)	Group III (N = 202)	p
Age (years)	60.0 ± 11.7	66.8 ± 11.6	52.4 ± 14.9	0.000^**^
Male	57 (30.5 %)	136 (61.3 %)	117 (58.2 %)	0.000^***^
Caucasian	105 (56.1 %)	145 (65.9 %)	138 (68.9 %)	0.000^***^
BMI (kg/cm^2^)	30.3 ± 6.2	28.6 ± 5.1	25.2 ± 2.6	0.000^**^
Waist circumference (cm)	98.3 ± 13.9	101.9 ± 13.5	95.6 ± 10.8^a^	0.004^*^
Diabetes mellitus	59 (32.6 %)	118 (53.4 %)	65 (32.5 %)	0.000^***^
Metabolic Syndrome	118 (64.1 %)	158 (71.8 %)	146 (73.0 %)	0.000^***^
Cardiovascular Disease	44 (24.6 %)	91 (41.9 %)	86 (43.7 %)	<.000^***^
o Cerebrovascular disease	20 (11.2 %)	26 (12.0 %)	21 (10.7 %)	0.912^***^
o Coronary disease	17 (9.5 %)	51 (23.5 %)	44 (22.3 %)	0.001^***^
o Peripheral vascular disease	11 (6.1 %)	18.9 (41.0 %)	46 (23.4 %)	0.000^***^
o Congestive heart failure	17 (9.5 %)	33 (15.3 %)	31 (15.7 %)	0.000^***^
Statin use	77/181 (40.7 %)	125/222 (54.3 %)	107/200 (53.2 %)	0.017^***^
Cholesterol(mM)	4.78 ± 0.93	4.65 ± 1.12	3.72 ± 1.00^a.b^	0.000^*^
HDL-C(mM)	1.27 ± 0.34	1.16 ± 0.33^c^	0.99 ± 0.37 ^a.b^	0.000^*^
LDL-C(mM)	2.78 ± 0.82	2.64 ± 0.97	1.89 ± 0.74^a.b^	0.000^*^
Triglycerides(mM)	1.61 ± 0.90	1.80 ± 0.98	1.91 ± 1.31	0.023^**^
Creatinine(μM)	73.4 ± 15.9	175.0 ± 69.8	930.8 ± 284.6	0.000^**^
ADMA (μM)	0.48 ± 0.12	0.75 ± 0.31^a^	1.34 ± 0.90	0.000^**^
CRP (mg/dL)	0.49 ± 0.73	0.62 ± 0.83	1.47 ± 3.78	0.004^**^

^*^ANOVA, ^**^Kruskal-Wallis-one way- ANOVA, ^***^Pearson chi-square

By post hoc Bonferroni test: ^a^groups III and II, ^b^groups III and I, ^c^groups II and I

HDL colestherol (HDL-C), LDL-C and triglyceride concentrations significantly decreased with decrease in estimated GFR. Plasma concentrations of ADMA and C reactive protein (CRP) significantly increased with decrease in estimated GFR.

The ε2 allele was present in 62 (10.3 %) patients, ε3 allele in 581 (96.2 %), and ε4 allele in 114 (18.9 %). The ε3/2 genotype was present in 57 (9.3 %) patients, ε3/3 genotype in 438 (71.7 %), ε4/2 genotype in 10 (1.6 %), ε4/3 genotype in 93 (15.2 %) and ε4/4 genotype in 13 (2.1 %). Table 2 depicts ApoE genotype and allele distribution among the 3 renal function groups. There was evidence of a significant association (*P* = 0.018) with Caucasians for the ε4 allele, when distribution of genotypes and alleles were stratified by race (Table 3).

**Table 2:** ApoE genotype and allele distribution among the 3 renal function groups

	Group I	Group II	Group III	Total	P*
	N (%)	N (%)	N (%)	N (%)	
ε3/ε2	17 (9.3)	19 (8.6)	16 (8.0)	52 (8.6)	NS
ε3/ε3	128 (69.9)	167 (75.2)	143 (71.9)	438 (72.5)	NS
ε4/ε2	7 (3.8)	1 (0.5)	2 (1.0)	10 (1.7)	NS
ε4/ε3	28 (15.3)	30 (13.5)	33 (16.6)	91 (15.1)	NS
ε4/ε4	3 (1.6)	5 (2.3)	5 (2.5)	13 (2.2)	NS
ε2 allele	24 (13.1)	20 (9.0)	18 (9.0)	62 (10.3)	NS
ε3 allele	173 (94.5)	216 (97.3)	192 (96.5)	581 (96.2)	NS
ε4 allele	38 (20.8)	36 (16.2)	40 (20.1)	101(18.9)	NS

*Pearson chi-square

**Table 3:** ApoE genotype and allele distribution based on race

	Caucasians	Afro-Americans	Others	Total	P^*^
	N (%)	N (%)	N (%)	N (%)	
ε3/ε2	36 (9.3)	8 (6.5)	10 (10.5)	54 (8.9)	NS
ε3/ε3	266 (68.9)	95 (77.2)	74 (77.9)	434 (71.9)	NS
ε4/ε2	6 (1.6)	3 (2.4)	1 (1.1)	10 (1.7)	NS
ε4/ε3	71 (18.4)	12 (9.8)	10 (9.5)	93 (15.4)	NS
ε4/ε4	7 (1.8)	5 (4.1)	1 (1.1)	13 (2.1)	NS
ε2 allele	42 (10.9)	11 (8.9)	11 (11.6)	64 (10.6)	NS
ε3 allele	373 (96.6)	115 (93.5)	94 (97.9)	582(96.2)	NS
ε4 allele	84 (21.8)	20 (16.3)	12 (11.6)	116 (19.2)	0.018

^*^Pearson chi-square

**Fig. 1:**
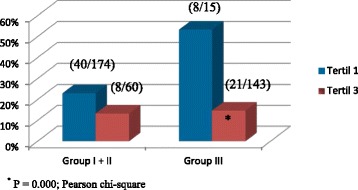
Comparison of frequency of ApoE ε4 allele between ADMA tertiles 3 and 1 in renal function groups

Table 4 depicts the statistically significant difference in plasma ADMA levels between patientes on renal replacement therapy (RRT) based on ApoE ε4 allele presence; Table 5 presents demographic and laboratory characteristics of patients according to ADMA tertile. ApoEε4 allele predominated in the first tertil of ADMA (Table 6). As also shown in Fig. 1, in group III, the frequency of ε4 allele of ApoE was significantly lower in the third tertile of ADMA compared with the first tertile. In groups I and II, there was no difference in allele frequency according to ADMA level. This association remained significant even after controlling for confounders (OR 0.329,95 % CI 0.155 – 0.699; *P* = 0.004), namely age, sex, BMI, DM, CVD, CRP, LDL-C and dialysis vintage (Table 7).

**Table 4:** Demographic and laboratory characteristics of patients according to RRT allocation and ApoE ε4 allele presence

	In RRT			Not in RRT		
	ε4 allele (+) (N = 40)	ε4 allele (−) (N = 158)	P	ε4 allele (+) (N =74)	ε4 allele (−) (N = 330)	P
Age (years)	52.47 ± 14.97	53.60 ± 14.36	0.669^*^	62.80 ± 13.36	63.81 ± 11.72	0.511^*^
Dialysis vintage	38.95 ± 24.24	37.22 ± 27.69	0.697^*^	-	-	
Male	25 (21.6 %)	91 (78.4 %)	0.574^**^	37 (19.7 %)	151 (80.3 %)	0.508^**^
Caucasian	31 (22.8 %)	105 (72.2 %)	0.403^**^	51 (20.6 %)	196 (79.4 %)	0.440^**^
BMI (kg/cm^2^)	25.15 ± 11.12	25.57 ± 2.44	0.356^*^	29.17 ± 6.40	29.33 ± 5.61	0.844^*^
Waist circumference (cm)	97.25 ± 15.96	99.30 ± 9.13	0.283^*^	99.81 ± 14.73	100.27 ± 13.59	0.798^*^
Diabetes mellitus	15 (23.1 %)	50 (76.9 %)	0.418^**^	32 (18.8 %)	138 (81.2 %)	0.895^**^
Metabolic syndrome	28 (19.4 %)	116 (80.6 %)	0.621^**^	48 (17.8 %)	222 (82.2 %)	0.733^**^
Cardiovascular disease	19 (22.6 %)	65 (77.4 %)	0.547^**^	26 (19.8 %)	105 (80.2 %)	0.653^**^
o Cerebrovascular disease	0 (0.0 %)	20 (100.0 %)	0.016^**^	13 (28.3 %)	33 (71.7 %)	0.073^**^
o Coronary disease	13 (29.5 %)	31 (70.5 %)	0.096^**^	11 (16.9 %)	54 (83.1 %)	0.703^**^
o Peripheral vascular disease	9 (20.5 %)	35 (79.5 %)	0.976^**^	10 (20.4 %)	39 (76.9 %)	0.697^**^
o Congestive Heart Failure	6 (19.4 %)	25 (80.6 %)	0.850^**^	7 (14.9 %)	40 (85.1 %)	0.480^**^
Statin use	23 (21.9 %)	82 (78.1 %)	0.551^**^	38 (19.4 %)	158 (80.6 %)	0.602^**^
Cholesterol(mmol/L)	3.75 ± 1.01	3.62 ± 0.90	0.470^*^	4.58 ± 1.06	4.75 ± 1.06	0.175^*^
HDL-C(mmol/L)	0.97 ± 0.34	1.08 ± 0.46	0.108^*^	1.15 ± 0.31	1.22 ± 0.35	0.128^*^
LDL-C(mmol/L)	1.92 ± 0.74	1.82 ± 0.76	0.469^*^	2.62 ± 0.85	2.79 ± 0.92	0.151^*^
Triglycerides(mmol/L)	1.98 ± 1.36	1.66 ± 1.14	0.160^*^	1.84 ± 1.06	1.69 ± 0.90	0.237^*^
ADMA (uM)	0.95 ± 0.45	1.44 ± 0.96	0.000^*^	0.58 ± 0.17	0.63 ± 0.28	0.134^*^
CRP (mg/dL)	1.67 ± 3.52	1.42 ± 3.88	0.711^*^	0.50 ± 0.72	0.57 ± 0.76	0.468^*^

^*^Student’s *t* test

^**^Pearson chi-square

**Table 5:** Demographic and laboratory characteristics of patients according to ADMA tertile

	First tertile (0.12 - 0.54) (N = 199)	Second tertile (0.55 - 0.79) (N = 206)	Third tertile (0.80 - 4.91) (N = 207)	P
Age (years)	62.37 ± 10.53	66.01 ± 11.95	65.64 ± 11.70	0.000^**^
Dialysis vintage (months)	2.48 ± 24.24	7.90 ± 1.48^c^	25.62 ± 1.92^a,b^	0.000^**^
Male	25 (21.6 %)	91 (78.4 %)	37 (19.7 %)	0.508^***^
Caucasian	31 (22.8 %)	105 (72.2 %)	51 (20.6 %)	0.440^***^
BMI (kg/cm^2^)	29.74 ± 5.55	29.11 ± 5.24	27.63 ± 13.43	0.000^**^
Waist circumference (cm)	99.46 ± 13.43	101.45 ± 11.40	101.88 ± 14.62	0.798^*^
Diabetes mellitus	15 (23.1 %)	50 (76.9 %)	32 (18.8 %)	0.895^***^
Metabolic syndrome	28 (19.4 %)	116 (80.6 %)	48 (17.8 %)	0.733^***^
Cardiovascular disease	66 (30.1 %)	77 (35.2 %)	76(34.7 %)	0.646^***^
o Cerebrovascular disease	0 (0.0 %)	20 (100.0 %)	13 (28.3 %)	0.073^***^
o Coronary disease	13 (29.5 %)	31 (70.5 %)	11 (16.9 %)	0.703^***^
o Peripheral vascular disease	22 (22.9 %)	35 (36.5 %)	39 (20.4 %)	0.697^***^
o Congestive heart failure	27 (34.2 %)	28 (35.4 %)	24 (30.4 %)	0.738^***^
Statin use	23 (21.9 %)	82 (78.1 %)	38 (19.4 %)	0.602^***^
Cholesterol(mM)	4.70 ± 1.08	4.45 ± 1.07^c^	4.59 ± 1.07^a.b^	0.000^*^
HDL-C(mM)	1.21 ± 0.34	1.05 ± 0.39	1.13 ± 0.36 ^a.b^	0.000^*^
LDL-C(mM)	2,71 ± 0.92	2.53 ± 0.88	2.24 ± 2.24 ^a.b^	0.000^*^
Triglycerides(mM)	1.99 ± 1.36	1.66 ± 1.14	1.84 ± 1.06	0.804^**^
Uric acid (mM)	0.36 ± 0.11	0.42 ± 0.12^c^	0.41 ± 0.10 ^b^	0.000^*^
Creatinine(μM)	131.72 ± 81.33	845.10 ± 239.56^c^	947.65 ± 290.84^a,b^	0.000^**^
CRP (mg/dL)	0.50 ± 0.08	0.66 ± 0.21	0.50 ± 0.72	0.321^**^

^*^ANOVA, ^**^Kruskal-Wallis-one way- ANOVA, ^***^Pearson chi-square

By post hoc Bonferroni test: ^a^ groups III and II, ^b^groups III and I, ^c^groups II and I

**Table 6:** ApoE genotypes and alleles among ADMA tertiles

	First tertile (0.12 - 0.54) (N = 199)	Second tertile (0.55 - 0.79) (N = 206)	Third tertile (0.80 - 4.91) (N = 207)	P^*^
ε3/ε2	19 (9.9 %)	21 (10.3 %)	16(7.8 %)	NS
ε3/ε3	123 (64.4 %)	147 (72.4 %)	158 (77.5 %)	NS
ε4/ε2	7 (3.7 %)	2 (1.0 %)	1 (0.5 %)	NS
ε4/ε3	37 (19.4 %)	30 (14.8 %)	25 (12.3 %)	NS
ε4/ε4	5 (0.8 %)	3 (0.5 %)	4 (0.7)	NS
ε2 allele	26 (13.6 %)	23 (11.3 %)	17 (8.3 %)	NS
ε3 allele	179 (93.7 %)	197 (97.0 %)	199 (97.5 %)	NS
ε4 allele	49 (25.7 %)	35 (17.2 %)	29 (14.2 %)	0.011
Adjusted residual	2.9	−0.7	−2.1	

^*^Pearson chi-square

**Table 7:** Logistic regression analysis of the association between ApoE ε4 allele frequency and third ADMA tertile, among patients in RRT

	OR	CI	p
Adjusted for ApoEε4 allele	0.329	0.155 - 0.699	0.004
Adjusted for ApoEε4 allele, age	0.330	0.155 - 0.700	0.004
Adjusted for ApoEε4 allele, age, race	0.341	0.162 - 0.719	0.005
Adjusted for ApoEε4 allele, age, race, sex	0.321	0.148- 0.697	0.005
Adjusted for ApoEε4allele, age, race, sex, BMI	0.317	0.146- 0.689	0.004
Adjusted for ApoEε4allele, age, race, sex, BMI, DM	0.263	0.111- 0.623	0.002
Adjusted for ApoEε4allele, age, race, sex, BMI, DM, CVD	0.270	0.114- 0.638	0.003
Adjusted for ApoEε4allele, age, race sex, BMI, DM, CVD, CRP	0.271	0.114- 0.640	0.003
Adjusted for ApoEε4allele, age, race, sex, BMI, DM, CVD, CRP, LDL-C	0.283	0.118 - 0.680	0.005
Adjusted for ApoE E4allele, age, sex, BMI, DM, CVD, CRP, LDL-C, dialysis vintage	0.283	0.118- 0.681	0.005

Analyses of clinical and laboratory characteristics of ε2 and ε3 alllele carriers showed no statistical difference between allele (+) versus allele (−) groups and stratified for RRT allocation or not (data not shown).

## Discussion

According to the literature, our data confirm that increased plasma ADMA levels are associated with decreasing GFR. We did not identify a difference in the distribution of ApoE genotypes and alleles between groups, but among patients on hemodialysis and with highest ADMA levels, the frequency of ε4 allele of ApoE was significantly lower when compared with that in patients in the first ADMA tertile. The observed difference did not occur in patients with normal renal function or in earlier stages of CKD.

In fact, ADMA levels are intrinsically related with renal function and this association has been studied in the past few years. The 3 groups in our study showed progressively higher plasma ADMA levels following the evolution of CKD. In accordance with our data, Fliser et al. found that ADMA was significantly associated with progression of nondiabetic kidney diseases [9]. Ravani et al. found that in patients with mild CKD plasma ADMA was inversely proportional to GFR and represented a strong and independent risk marker for progression to end stage renal disease (ESRD) and mortality [10]. Initially, the elevation of ADMA in uremia was credited only to the decrease in GFR. Thereafter, elevations in plasma ADMA concentrations were also reported in incipient renal diseases, such as autosomal-dominant polycystic kidney disease [11] and IgA nephropathy [12], and in normal renal function such hypercholesterolemia or atherosclerosis [13]. ADMA is renally excreted to some extent, but its rise in CKD is due to increased activity of protein arginine methyltransferases [14], a family of enzymes involved in the process of post-translational methylation of arginine residues, a common mechanism of protein modification. In addition, accumulation of ADMA in patients with CKD is also related to decreased activity of the enzyme dimethyl arginine dimethyl aminohydrolase (DDAH) [14], part of the main pathway for ADMA degradation, which hydrolyzes ADMA to dimethylamine and L‐citrulline. So far, two isoforms of DDAH have been characterized and cloned: DDAH I is predominately found in tissues that express neuronal NOS, whereas DDAH II is predominately found in tissues expressing endothelial NOS [14, 15]. It is estimated that healthy humans produce approximately 300 μmol of ADMA per day, of which approximately 250 μmol are metabolized by DDAHs [16].

ADMA is an endogenous inhibitor of nitric oxide synthases (NOS), which may in part explain the impaired vasorelaxation, elevated inflammation, and reduced angiogenesis reported in CKD patients and animal models of CKD [17]. The role of ADMA in atherogenesis is not restricted to its inhibitory action on NOS. It upregulates the expression of acyl-coenzyme A cholesterol acyltransferase 1 present in macrophages and is implicated in the formation of foam cells [7]. ADMA also impairs the migratory capacity of angiogenic progenitor cells (APCs) in patients with coronary artery disease through a micro RNA-21-dependent mechanism, inhibiting superoxide dismutase 2 in APCs [18].

Accumulation of ADMA is a risk factor and is causally related to the development of endothelial dysfunction and cardiovascular disease in patients with CKD [19–22]. Zoccali et al. demonstrated in patients undergoing hemodialysis with initially normal carotid intima-media thickness, that ADMA and CRP are interacting factors in the progression of carotid intimal lesions [23].

ApoE gene contains three potential alleles: ∈2, ∈3 and ∈4, forming six genotypes and determining diversity in clinical expression. Allele frequency from high to low is ε3, ε2 and ε4, and ε3ε3 is the most common phenotype in humans [24], as seen in our study. In NHANES III [25], the ε2 allele was the rarest for each ethnicity examined, and the frequency of the e4 allele was 10.8 % among Mexican Americans, compared to 15 % in whites and 22 % in non-Hispanic blacks. Similarly to Chu et al. [25], we demonstrated that the ε2 allele was the less prevalent in all groups in our analysis of allele and genotype distribution by race. ApoE ε4 allele showed evidence of significant association with Caucasians.

Several previous studies reported that the ε2 allele is a genetic risk factor for all-cause CKD [26] and related to increased risk of ESRD. [27, 28] In contrast, the ε4 allele may be associated with a lower risk of diabetic nephropathy [29]. Furthermore, lipoprotein glomerulopathy, a rare inherited renal disease characterized by proteinuria and progression to CKD, has a strong relation with mutations in the ApoE gene [30]. An association between ESRD and ApoE polymorphism has been found in some but not all studies. Hubacek el al. found more carriers of the ε*2* allele in ESRD patients (15.9 %) than in controls [28]. However, Roussos et al. [31] found that patients with ESRD showed no difference in ε2, ε3 and ε4 distribution compared with the control group. Feussner et al. also failed to find an association between ε2 and ESRD. [32] We did not find statistical differences between groups stratified by renal function, analyzing allele and genotype distribution.

When we accessed the relation between ADMA level and ApoE allele frequency among those patients in RRT, we observed that patients on hemodialysis and with the highest ADMA levels exhibited a significantly lower frequency of the ε4 allele when compared with that of patients in the sum of first and second tertiles of ADMA. This association remained significant even after correction for confounding factors. An interrelationship of ADMA and ApoE in atherogenesis was assessed in the work of Jacobi et al., in which mice defficient in ApoE and overexpressing DDAH showed reduced plaque formation in the aorta. A functional analysis of aortic ring preparations revealed improved endothelial function in mice overexpressing DDAH [33]. This association is interesting because it provides the patient with potentially greater endothelial dysfunction, represented by higher ADMA, and risk of cardiovascular complications the potential protection associated with the absence of the ε4 allele. Medina-Urrutiaet al. [34] showed that the presence of the ε4 allele was associated with elevations in LDL-C, while the presence of ε2 was associated with decreased levels of LDL-C. Compared with individuals with the ε*3*/ε*3* genotype, ε*2* carriers have a 20 % lower risk of coronary heart disease and ε*4* carriers have a slightly higher risk [35]. A recent study by our group in a cohort of hypertensive patients with metabolic syndrome, revealed a higher prevalence of CVD among ∈4 allele carriers [36]. On the other hand, this association suggests that uremia is capable of determining lower plasma ADMA levels among ε4 allele carriers. ApoE ε4 allele expression could be limited by the presence of certain clinical variables, as can be seen in our study.

This study had some limitations. First, it was a cross-sectional study. The results of a study composed exclusively of hypertensive patients, conducted at a single center, cannot be extrapolated to the general population. It is descriptive and the mechanisms underlying this association cannot be inferred here. A greater number of participants in each renal function group could have allowed us to determine if this association between lower frequency of ApoE ε4 allele with higher level of ADMA also occurs in earlier stages of CKD. Genetic analysis methods (single nucleotide polymorphism) have limitations in the comprehension of the genotype - phenotype interface: these methods do not enable the simultaneous analysis of multiple genes, like in genome wide association, hampering the search for a genetic signature for polygenetic diseases.

## Conclusions

The presented results show, for the first time, that uremia is capable of determining lower plasma ADMA levels in hypertensive ε4 allele carriers. This association between lower frequency of ApoE ε4 allele and higher ADMA levels in ESRD may indicate a new way to approach atherogenesis in the inflammatory state of uremia. Confirmation of such observations needs to be validated in a longitudinal observation with a larger population study.

## Methods

The study was approved by the Ethics and Research Committee (no. 311.413/06.21.2013, Ministry of Health, Brazil). All patients received relevant explanations and signed Ethics and Research Committee Informed Consent.

Six hundred twenty patients were stratified into 3 groups according to estimated GFR by CKD-EPI formula: group I > 60 mL/min, group II ≤ 60 mL/min and > 15 mL/min, and group III ≤ 15 mL/min or in hemodialysis. Exclusion criteria were as follows: aged under 18 years, clinical or laboratory suspicion of acute renal failure, pregnancy and cancer.

Patient’s data were collected by clinical evaluation, physical examination, file reviews and laboratory tests. Presence of CVD was considered when identified in office or registry files: history of stroke, peripheral vascular disease, coronary artery disease or CHF. [37] Identification of DM was determined according to the American Diabetes Association Guidelines [38].

Plasma concentrations of glucose, total cholesterol and triglycerides were determined by automated enzymatic assays. HDL was measured in serum by the homogeneous method after precipitation of VLDL and LDL with phosphotungstate and magnesium ions, centrifugation and measurement of the supernatant absorbance at 500 nm wavelength, following the manufacturer’s instructions. LDL was calculated according to the Friedewald equation. Renal function was assessed by plasma urea level, measured by colorimetric-enzymatic assay and by plasma creatinine level, determined by the Jaffé method with calibration traceable to an isotope dilution mass spectrometry reference measurement procedure [39]. CRP was measured by ultrasensitive immunoturbidimetry, following the manufacturer’s instructions.

Blood samples from subjects with ESRD undergoing hemodialysis was always obtained immediately before treatment.

### ADMA levels

Plasma ADMA levels were measured by HPLC, as describedby Teerlinket al. [40] Briefly, samples were prepared as follows: 200 μL of plasma containing EDTA or heparin were transferred to an Eppendorf tube (1.5 mL), and 100 μL of internal standard solution (40 μM monomethylarginine) were then added. PBS was added to complete the volume to 1 mL. This mixture was introduced into an OASYS extraction cartridge (Waters) coupled to a vacuum system previously equilibrated with 1 mL of methanol and 1 mL of deionized water. Next, the cartridge was rinsed with 1 mL of 100 mM HCl, followed by 1 mL of methanol, to elute neutral compounds and acids, and elution was performed with 1 mL of ammonia/water/methanol (10/40/50) solvent. The eluate recovered was dried at 60 °C in a speed-vacuum system, and the residue obtained was dissolved in 100 μL of water, followed by the addition of 100 μL of ortho-phthaldialdehyde. After 15 min of reaction, the samples were transferred to appropriate HPLC vials.

We used a Symmetry C18 column (3.9 x 150 mm; 4 μm) coupled to a pre-column equilibrated with the same stationary phase. The mobile phase A consisted of 50 mM potassium phosphate buffer (pH 6.5) and mobile phase B (acetonitrile/water; 1/1, v/v). Samples (20 μL) were separated using an HPLC system with automatic injector. Standard solutions containing arginine (25, 50, 75, 100 and 150 μM) and ADMA (0.25, 0.50, 1.00, 2.5, 5.0 μM) and 40 μM of internal standard solution were extracted as described above to be injected before and after the injection of samples. The flow rate was 1.1 mL/min.

The time interval between each injection was 30 min, and fluorescence was measured at emission and excitation wave lengths of 340 and 455 nm, respectively.

### Analysis of ApoE gene polymorphisms

The sequence of 244 base pairs of the ApoE gene was amplified by PCR, using the primers: *sense* 5’-TCCAAGGACCTGCAGGCGGCGCA-3’ and *antisense* 5’ACAGAATTCCGCCCCGGCCTGGTACACTGCCA-3’.

The PCR products were digested with the restriction enzyme *Hha*I, and the fragments were separated by 10 % polyacrylamide gel electrophoresis. Afterwards, the gel was incubated with ethidium bromide (0.5 μL/mL) for 90 min at 120 V in 1xTBE solution, and the DNA fragments were visualized with UV light. The ε2, ε3 and ε4 alleles were analyzed and the corresponding genotypes determined.

Groups were analyzed and compared with regard to epidemiological, clinical and laboratory characteristics as well as ApoE allele and genotype distribution. The prevalence of different alleles and genotypes were analyzed in relation to race, where Hardy-Weinberg equilibrium was observed. Plasma ADMA levels were compared on the basis of RRT allocation ApoE polymorphism.

### Statistical analysis

Results were expressed as mean ± SD or percentage. Comparisons of continuous variables among the three renal function groups were carried out with ANOVA and Kruskal-Wallis-one way-ANOVA, followed by *post hoc* Bonferroni tests. Comparisons of frequency between groups were performed using Pearson chi-square test. Binary logistic regression analysis was used to test the association between ApoE ε4 allele frequency and endothelial dysfunction, in patients in RRT. The level of nullity was fixed at 0,05 or 5 % for all tests.

## Abbreviations

ADMA: Asymmetric dimethylarginine
NOS: Nitric oxide synthase
ApoE: Apolipoprotein E
LDL-C: LDL cholesterol
GFR: Glomerular filtration rate
CKD-EPI: Chronic kidney disease epidemiology collaboration
PCR: Polymerase chain reaction amplification
HPLC: High performance liquid chromatography
CVD: Cardiovascular disease
CKD: Chronic kidney disease
CHF: Congestive heart failure
NO: Nitric oxide
DM: Diabetes mellitus
CRP: C reactive protein
RRT: Renal replacement therapy
ESRD: End stage renal disease
DDAH: Dimethyl arginine dimethyl aminohydrolase
APCs: Angiogenic progenitor cells
